# In Vivo Experimental Study of Noninvasive Insulin Microinjection through Hollow Si Microneedle Array

**DOI:** 10.3390/mi9010040

**Published:** 2018-01-20

**Authors:** Drago Resnik, Matej Možek, Borut Pečar, Andrej Janež, Vilma Urbančič, Ciprian Iliescu, Danilo Vrtačnik

**Affiliations:** 1 Laboratory of Microsensor Structures and Electronics, Faculty of Electrical Engineering, University of Ljubljana, Tržaška 25, SI-1000 Ljubljana, Slovenia; matej.mozek@fe.uni-lj.si (M.M.); borut.pecar@fe.uni-lj.si (B.P.); danilo.vrtacnik@fe.uni-lj.si (D.V.); 2 Department of Endocrinology, Diabetes and Metabolic Diseases, University Medical Centre Ljubljana, Zaloška 7, SI-1000 Ljubljana, Slovenia; andrej.janez@kclj.si (A.J.); vilma.urbancic@kclj.si (V.U.); 3 National Institute for Research and Development in Microtechnologies, IMT-Bucharest, Bucharest 077190, Romania; ciprian.iliescu@imt.ro

**Keywords:** hollow Si microneedle array, microinjection, in vivo insulin delivery, drug transfer efficiency

## Abstract

An experimental study of in vivo insulin delivery through microinjection by using hollow silicon microneedle array is presented. A case study was carried out on a healthy human subject in vivo to determine the influence of delivery parameters on drug transfer efficiency. As a microinjection device, a hollow microneedle array (13 × 13 mm^2^) having 100 microneedles (220 µm high, 130 µm-outer diameter and 50 µm-inner diameter) was designed and fabricated using classical microfabrication techniques. The efficiency of the delivery process was first characterized using methylene blue and a saline solution. Based on these results, the transfer efficiency was found to be predominantly limited by the inability of viable epidermis to absorb and allow higher drug transport toward the capillary-rich region. Two types of fast-acting insulin were used to provide evidence of efficient delivery by hollow MNA to a human subject. By performing blood analyses, infusion of more-concentrated insulin (200 IU/mL, international units (IU)) exhibited similar blood glucose level drop (5–7%) compared to insulin of standard concentration (100 IU/mL), however, significant increase of serum insulin (40–50%) with respect to the preinfusion values was determined. This was additionally confirmed by a distinctive increase of insulin to C-peptide ratio as compared to preinfusion ratio. Moreover, we noticed that this route of administration mimics a multiple dose regimen, able to get a “steady state” for insulin plasma concentration.

## 1. Introduction

Transdermal drug delivery (TDD) is an attractive alternative to subcutaneous route of delivery for various drugs and vaccines, but usually limited by the extremely low permeability of the outer skin layer, a 10–15 μm thick *stratum corneum* (SC). Microneedle arrays (MNA) have shown immense potential to effectively deliver drugs intradermally and transdermally, particularly when a relatively small volume of drug is required [1,2]. One of the desired benefits of MNA is to replace, where appropriate, conventional hypodermic needles, avoiding anxiety and/or pain to the patient. The advantages and disadvantages of the transdermal route for drug delivery by using microneedles have been thoroughly presented and discussed in numerous review papers [3,4,5,6,7,8]. In addition, different strategies to facilitate this delivery route were developed and proposed [3,9,10,11,12,13]. In this direction two main categories of MNA were developed: solid MNA and hollow MNA. Solid MNA, which were in the past most often made of nondegradable materials such as metal [14], silicon [15], or polymers [16] with aim to facilitate the passive drug diffusion by disrupting the SC layer, are being recently replaced by various dissolving, biodegradable materials. Different materials were investigated for the fabrication of dissolving microneedles such as starch-gelatin dissolving microneedle patches loaded with insulin [17], microneedles made of hydrocolloidal pectin [18], a polylactic acid-a biodegradable polymer [19] or hyaluronic acid, representing both a base material and the material to improve the transdermal permeability for relatively high molecular weight drugs [20]. Their results proved to be promising in terms of delivery efficiency, patient safety and waste management.

An important advantage of hollow over solid microneedles is the possibility to facilitate pressure-driven liquid flow across the skin. The dose and flow rate can be therefore precisely controlled [21]. Hollow MNA were successfully used for diagnostic extraction and sampling of biological fluids by capillary action [22,23,24]. Furthermore, with the advance of microfluidics and microneedle technologies, the development of delivery and analyses devices and systems on a single chip can be realized for diagnostics and treatment purposes [21].

Beside Si material, hollow MNA fabricated from other materials have gained interest in recent years such as SU8 polymer [24,25,26], clay reinforced polyimide [27], or metal electroplated polymer MNA [28]. Early studies related to delivery through hollow needles are reported by Martanto et al. [29] by using a single hollow glass microneedle inserted 1 mm deep into the human cadaver skin for delivery of sulforhodamine solution and by Sivamani et al. [30] who provided evidence of successful transdermal delivery of methyl nicotinate on human subjects in vivo by using hollow microneedles.

The hollow MNA requires an appropriate interface to provide substance delivery and enable proper handling for practical use. One of the main problems is how to maintain a consistent contact between MNA and the skin, which may strongly affect targeted dose. Therefore, appropriate approaches to provide firm, leak-free interface during delivery period are mandatory.

Among different drug formulations, insulin delivery using this approach is very desirable for diabetic patients. Subcutaneous administration of insulin by means of hypodermic needles, insulin pens and catheters connected to insulin pumps currently still remain prevalent methods due to their reliable therapeutic effect. Inhaled insulin is another alternative but has certain limitations and still requires a combination of basal insulin through traditional methods [31]. Most of the studies dealing with insulin delivery by hollow MNA were performed on rodents with induced diabetes [32,33,34,35]. Gupta et al. [31] reported the first proof of concept that single hollow glass microneedles can effectively deliver bolus insulin to type 1 diabetes subjects in a minimally invasive manner, but penetrating to the depth of 1–5 mm, which cannot be considered as pain-free approach.

Here, we report on a complete solution for in vivo noninvasive delivery of insulin through a hollow microneedle array. In order to avoid pain and patient discomfort, to provide sufficient tradeoff between penetration efficacy and robustness of microneedles, and by taking into account the microfabrication constraints, the height of the Si hollow microneedles was established in the range between 200 and 220 µm. The main delivery parameters (flow rate, dose, applied force on the MNA, MNA size, anatomical delivery site) were analyzed in order to explain the limitation of the process and the transfer efficiency (rate-limiting factors). As a result, we established a delivery protocol that assured a reasonable transfer efficiency of the delivered substance. In order to demonstrate and evaluate the system drug transfer efficiency, in the first stage, the leak that can appear from the needle-skin interface was evaluated using methylene blue (MB) dye. In the second stage, saline solution was delivered into the skin to establish the transfer efficiency. Finally, noninvasive delivery of insulin through hollow MNA was performed on the healthy human subject. Blood analyses at predefined time intervals were carried out to analyze glucose, insulin and C-peptide levels after the insulin delivery. The pharmacokinetic profile of the insulin through hollow microneedles in this case showed a relatively constant plasma concentration over time and supported the similarity with the “drug steady state” principle in multiple doses regimen. This is to our knowledge one of very few studies presenting the results of noninvasive, in vivo insulin microinjection to human subject by using hollow MNA and also revealing specific limitations of the delivery method.

## 2. Materials and Methods

### 2.1. Design and Fabrication of MNA

**Figure 1:**
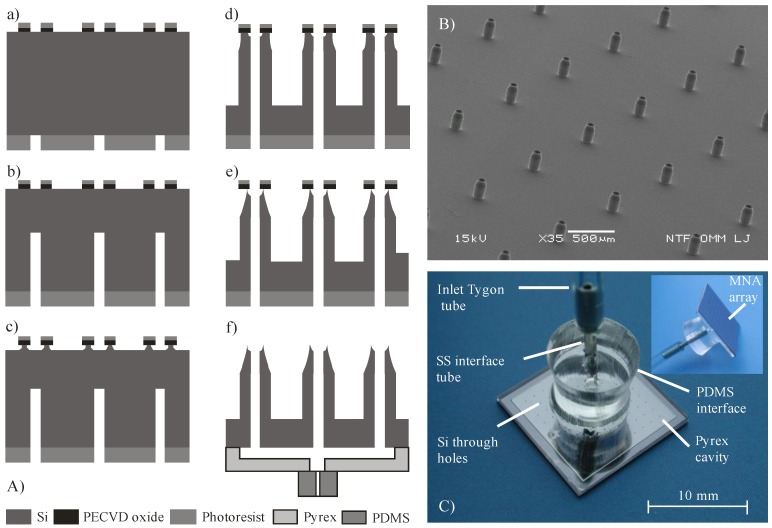
(**A**) Fabrication process steps: (**a**) front–to-back aligned circular openings; (**b**) back side directional DRIE; (**c**) front side isotropic; ICP assisted DRIE; (**d**) directional front side DRIE; (**e**) isotropic DRIE sharpening; (**f**) anodic bonding of Pyrex reservoir and covalent bonding of PDMS fluidic inlet; (**B**) SEM micrograph of fabricated hollow MNA with sharpened apex, 220 µm high, interdistance 1 mm, base diameter 130 µm; (**C**) Assembled MNA with 100 microneedles. View from the rear side, showing fluidic connection, distribution cavity and through holes. Inset shows partially front side and fluidic connection.

Experimental work consisted of design and fabrication of hollow Si MNA, a Pyrex glass reservoir, anodic bonding of previous mentioned chips, and appropriate assembly for delivery and handling and setting up the measuring system for the evaluation of delivery method. Silicon microneedle arrays with outer dimensions of 13 × 13 mm^2^ with 100 microneedles spaced in grid 1.0 × 1.0 mm^2^ were designed and fabricated on double side polished 100 mm silicon wafers, N-type, resistivity of 10 Ω·cm and thickness of 400 μm. The main fabrication steps are presented in Figure 1A. Plasma-enhanced chemical vapor deposition (PECVD) of 3 μm thick silicon oxide of was first performed to provide an etch mask for the front side deep reactive ion etching (DRIE). To obtain the patterned etching mask on the front side, oxide dots with outer diameter of 130 μm and inner diameter of 50 µm, which will represent shaft and lumen of the microneedle, respectively, were first patterned by 1.8 μm-thick positive photoresist HPR 504. The PECVD silicon oxide was patterned by reactive ion etching (RIE) using a classical CHF_3_/O_2_ process. Then, backside deposition (PECVD SiO_2_-3 µm-thick) and patterning was performed using an 8 μm-thick AZ 9260 photoresist. This process provided an etch mask for the realization of through holes during backside DRIE process.

Silicon etching was performed in a DRIE system Plasmalab 100 ICP180. First, back side etching (240 µm in depth) of holes (microneedle lumen) was performed using a classical Bosch process. This was followed by the front side fabrication of microneedle shafts and through holes. First part of etching was performed by a combination of inductively coupled plasma (ICP), isotropic etching and Bosch process to obtain a tapered shaft and simultaneous sharpening of the top by underetching the PECVD oxide mask. In the next step, an anisotropic dry etching process was performed in order to reach the required shaft height and meet the inner lumen with the backside prefabricated hole. For the etching of a 220 μm high microneedle shaft, the lumen was etched to the depth of only 160 μm, thereby reaching the hole resulting from the previous backside process. The reason that hole was etched slower than the shaft originates from the effect known as aspect ratio dependent etching (ARDE) [36].

An image with the processed hollow MNA is presented in Figure 1B. The hollow MNA have also, beside the sharp circular apex, a tapered upper part of the shaft and should therefore require lower penetration force compared to similar design presented by Chua et al. [23]. The microneedle shafts are relatively widely spaced (1 mm) with respect to their height (220 µm), which assured constant etching rate of microneedle shafts (1 μm/min). From the application point of view, this is also beneficial to reduce the “bed of nails” effect [37]. Etched profiles and resulting geometry were measured by optical microscopy and scanning electron microscopy (SEM, JSM-5610F, JEOL, Tokyo, Japan).

In order to enable uniform liquid distribution to the entire array of microneedles, fluidic access to individual microneedles was realized via a common glass reservoir attached onto the rear side of Si MNA chip, as can be seen in Figure 1C. A Pyrex glass reservoir was fabricated separately by wet micromachining similar to the process described in [38]. Recessed structure was etched 85 µm deep into 700 µm Pyrex glass via a patterned Cr/Au mask layer in 49% HF, representing a reservoir with volume of 100 µL. Next, an inlet hole was mechanically drilled in the center of a glass. Attachment of the fabricated glass reservoir to the rear side of the silicon microneedle array was accomplished by anodic bonding. As could be seen from Figure 1C, only a 1 mm wide rim is hermetically sealing the reservoir. To provide reliable and robust fluidic connection, a PDMS interface with a central hole was covalently bonded to the glass. Figure 1C shows the assembled hollow MNA with rear side fluidic connection ready for further experimental work. A Tygon S-50-HL tube, declared as biocompatible, was used for connecting MNA to a syringe pump (TSE Systems, model No 540060-HP). Dicing the 10 × 10 MNA Si chips and adapting the described assembly procedure also enabled preparation of smaller 3 × 3 MNA.

It is well known that a damaged or broken microneedle represents low flow resistance, i.e., a weak spot where most of the leaking during delivery process occurs. In order to detect and avoid such malfunctions, each MNA was optically inspected prior to the bonding process step. Microneedle lumens were inspected by light transparency and microneedle shafts and apexes were inspected by stereo zoom microscopy at tilted view. Similar method to determine clogging of the lumens was used in [25] for their metallic hollow MNA. Prior to use, the cleaning of assembled MNA (Figure 1C) usually comprised ultrasound agitation in ethanol, followed by deionized water (DI) rinse and N_2_ drying. The post-fabrication yield was between 60 and 75% and the post-assembly yield was between 50 and 60%.

### 2.2. MNA Carrier and Skin Preparation

Attachment approach may strongly influence the delivery process by e.g., non-uniform distribution of pressure exerted by MNA on the skin. During the drug delivery interval patient activity, muscle contraction, skin tension or sweating are also expected to influence the skin-MNA interface behavior. This may lead to a change in penetration depth, pronounced leak of delivered substance and finally the loss of targeted dose control. Different approaches to how to apply and attach the MNA were examined during this study such as fastening by elastic strap, adhesive patch attachment, application of constant force, vacuum and other. Shear stress can be induced on the skin when MNA is not properly aligned. A cushioning effect of subcutaneous fat and muscle layers somewhat mitigate this effect [34].

**Figure 2:**
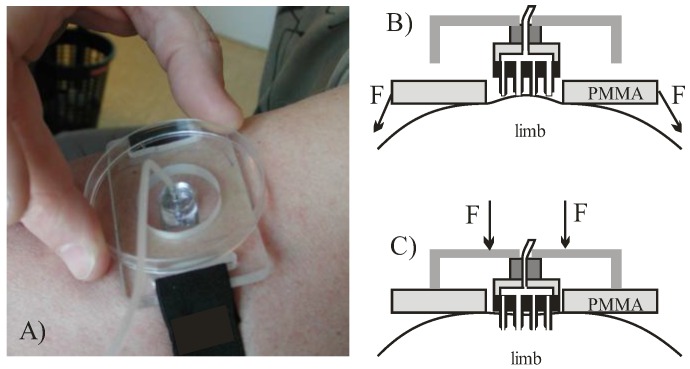
(**A**) MNA carrier and pre-stretching fixture; (**B**) formation of a bulge and application of MNA on pre-stretched skin; (**C**) skin penetration by MNA and delivery.

A special MNA carrier and a pre-stretching belt were designed in order to reduce the number of significant variables affecting the repeatability (Figure 2A). Skin was pre-tensioned by fastening first the poly(methyl methacrylate) (PMMA) fixture (shown in Figure 2B). By pressing a fixture onto the skin a hemispheric bulge was formed with confined circular edge and stretched skin in central part, which provided a site onto which the square MNA chip was then pressed. The described attachment approach is illustrated in Figure 2 and was used in all the following experiments.

To uniformly access the skin and in particular to allow better control of orthogonality during microneedle penetration, MNA was fixed on the additional larger PMMA planar fixture, serving also as an adjustable penetration depth limiter (Figure 2C). During penetration the central part of the MNA came into contact with the skin first. By increasing the force on the PMMA fixture the last to touch the skin are the microneedles at the corner sites. This reduces the viscoelastic effect of skin recession under the central part of the MNA due to pressure build-up during delivery phase. After each use, the MNA carrier was disassembled, the MNA was cleaned by mild alkaline solution, rinsed by pressurized DI water and dried by N_2_. MNA were then optically inspected for eventual clogging, and reused.

### 2.3. Saline and Insulin Delivery Protocol

Delivery protocol was first established empirically for saline delivery by MNA and then maintained throughout the experiments to minimize error due to manipulation variations. In the loading step by aid of syringe pump, the liquid has to provide the complete air removal from the reservoir and microneedle lumens. Partially flooded front MNA surface was then pressed to the skin and a certain amount of substance inevitably remained in the space between skin and penetrating MNA. This may increase the error of transfer efficiency determination when collecting the residual liquid left on the skin after delivery and was the main source of inaccuracy of transfer efficiency evaluation method, presented in Section 3.2.1. In the drug release step, when MNA was attached to the skin by aid of fixture (Figure 2) a syringe pump with predefined flow rate started to deliver liquid substance continuously through the MNA-skin interface. The same delivery protocol was also used for insulin delivery tests by MNA on a human subject in vivo. Tests were carried out on the healthy Caucasian male volunteer, age 55, with his written consent. The study (project identification code L2-4122) was conducted in accordance with the Helsinki declaration (1975) and in accordance with the internal protocol of medical center. Preinfusion reference blood samples were taken 10–15 min prior to insulin delivery. Before insulin delivery the subject fasted for 10 h. After completing the insulin delivery blood samples were acquired periodically at 15 min, 30 min, 45 min, and every 30 min thereafter up to 2 h according to established protocol. Blood from the vein on the dorsum of the hand was drawn into a test tube (3 mL) each time without additives and transported immediately to the laboratory where it was assayed for the glucose level, insulin and C-peptide by Chemiluminesce Immuno Assay (CLIA) method.

## 3. Results

### 3.1. Methylene Blue Dye Delivery into the Skin by MNA

**Figure 3:**
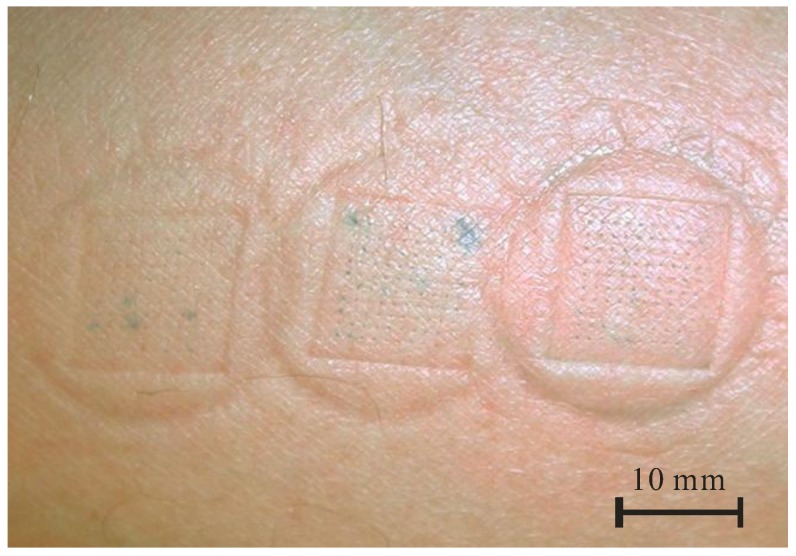
Methylene blue dye delivery by hollow MNA at ventral forearm (flow rate 50 µL/h, delivery time from left to right: 1 min, 2 min and 3 min).

In order to optically determine the uniformity of liquid flow through an array of 100 microneedles and to detect the eventual failure sites, preliminary tests were performed with MB dye delivery into the human arm skin, often used for microneedle penetration studies [23,39]. Figure 3 shows stained penetration sites after the consecutive MB delivery for three different delivery times, i.e., 1 min, 2 min and 3 min, from left to right, respectively. For such small doses, no leak was observed and the individual sites were clearly marked, showing uniform distribution of dye through each of 100 hollow microneedles. In Figure 3, a blotch due to the pressed device clearly shows a pressure distribution on the skin and increased intensity of stained spots with delivery time. The outer, circular form originated from a fixture that forms skin bulge (Figure 2B) and inner, squared form was due to the Si chip edge (Figure 2C). No skin irritation at the stained sites was observed after this treatment and the blotches normally disappear after 10–20 min. Optical inspection revealed that no mechanical damage occurred on MNA, thus confirming the robustness of microneedles.

### 3.2. Saline Delivery into the Skin by MNA

In order to minimize the number of the in vivo experiments with insulin, saline solution delivery was employed for initial stage of in vivo experiments. The purpose of this approach was to establish the appropriate range of delivery parameters, which would be applied for the insulin delivery in the final experiments. Delivery of isotonic saline (0.9 wt % NaCl ionic solution in deionized water) could not be taken directly analogous to delivery of macromolecules of insulin, but since the water is a transport solute and concentrations of both are low, the results could be taken as the first approximation and therefore provide useful guidelines [40].

#### 3.2.1. Transfer Efficiency *η*

To set a quantification criterion, the transfer efficiency *η* was defined as the ratio between the amount of the substance delivered through the skin and the total amount of substance delivered by the syringe pump (Equation (1)). The amount of delivered substance through the skin was determined indirectly by the gravimetrical method. The analytical scale (Kern, ABJ, resolution 0.1 mg) was used for this purpose. Immediately after delivery, the MNA was removed from the skin and the residual liquid left on the skin was absorbed by highly absorptive material and weighted. Since the mass of absorptive material was weighed prior to this, the amount of residual substance could be determined and subtracted from the total amount of liquid delivered by the syringe pump.

(1) $$\eta =\frac{m_{p}-\left(m_{w}-m_{c}\right)}{m_{p}}\times 100\;(\%)$$

where *m_p_* is the mass of delivered substance by the syringe pump (dispensed volume times density), *m_w_* is the mass of the water absorptive material together with absorbed residue and the *m_c_* is the mass of the absorptive material. Once residual liquid was collected no surplus out-diffusion from the skin was observed.

The collecting procedure was maintained in a repeatable manner to avoid inconsistencies and minimize the error between the tests. The vaporization of the substance during acquisition and weighing period was also taken into account. After performing more than a hundred tests and residual sample acquisitions, it was empirically determined that the accuracy of the method was better than ±10%.

#### 3.2.2. The Influence of Flow Rate and MNA Array Size on Transfer Efficiency

Array size and flow rate are considered to have significant impacts on the transfer efficiency at least from three points of view. First, taking into account the number of delivery channels (large number of independent sources); second, delivery distribution across larger skin area where absorption and diffusion can take place simultaneously; and third, due to pressure distribution acting on skin surface. The pressure by which the individual microneedle of large array 10 × 10 acts on the skin during penetration is nearly an order of magnitude lower compared to smaller array of 3 × 3 microneedles at equal applied force.

**Figure 4:**
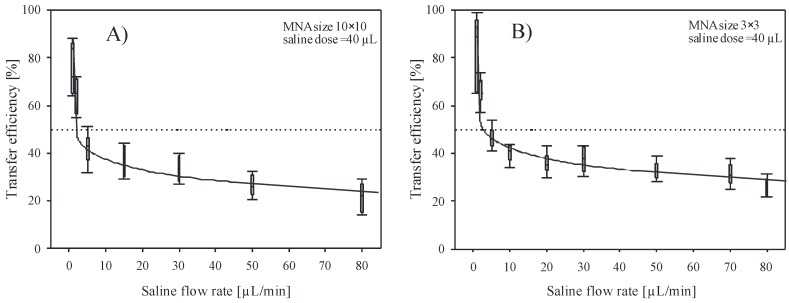
Transfer efficiency versus flow rate of saline solution delivered into the skin by (**A**) 10 × 10 and (**B**) 3 × 3 array of hollow microneedles. Cumulative dose was maintained constant (40 µL) for each flow rate.

Figure 4A,B show dependency of the transfer efficiency versus saline flow rate during delivery by 10 × 10 and 3 × 3 MNA. Delivery site was the upper arm. Flow rate varied between 1 and 80 µL/min and the dose in each of these experiments was maintained constant at 40 µL. Applied force on the MNA was held at 3 ± 0.5 N. In Figure 4A,B, each flow rate bar represents at least 6 independent measurements. As shown, the transfer efficiency decreases rapidly by increasing the flow rate above 5 µL/min. High transfer efficiency (>80%) was obtained for flow rate of 1 µL/min, which was the lowest flow rate applied during experimental work and decreased to 10–20% for flow rates exceeding 50 µL/min. Lower flow rates (<1 µL/min) require excessive delivery times and were found difficult to control due to dynamic behavior of the skin-MNA interface. In addition, patient discomfort due to long delivery time and vaporization of residual liquid, which reduces the measurement accuracy, were the reasons to omit lower flow rates. For instance, Roxhed et al. [39] reported the presence of excess liquid on the skin after delivering dye with flow rate of 3.3 µL/min. They assumed that already this flow rate was too high for the skin tissue to fully absorb the liquid.

Results of experimental work with small 3 × 3 arrays are presented in Figure 4B and show very similar behavior, but consistently higher transfer efficiency values. Low saline flow rates reached high transfer efficiency (>90% for 1 µL/min) and then nearly exponentially decreased toward 22–32% for flow rate of 80 µL/min, which was the highest flow tested with small array. By comparing the results shown in Figure 4 for two array sizes, smaller array size shows minor overall improvement of transfer efficiency, estimated to be within 5–8%. A bed of nails effect could be one of the reasons for the obtained difference between the two array sizes. For the 3 × 3 array, larger pressure on each microneedle could improve the penetration across the skin. It is assumed that the latter dominates over the fact that in the case for the 10 × 10 array the delivery across larger skin area is taking place. For the 10 × 10 array, an order of magnitude larger skin area is available for the substance distribution. However, taking into account similar applied force, only one tenth of the pressure is acting on each microneedle, thus the insertion depth might be reduced. As shown by the error bars, the distribution of individual measurements is scattered, indicating that repeatability of measurements can also be attributed, besides the evaluation method inaccuracy, to ambient and human skin condition variations during a more than one year period of experimental work. Regardless of the large tolerances of measured results, very indicative trend confirms the significant influence of flow rate on transfer efficiency in both cases.

#### 3.2.3. Influence of Delivered Dose on Transfer Efficiency 

**Figure 5:**
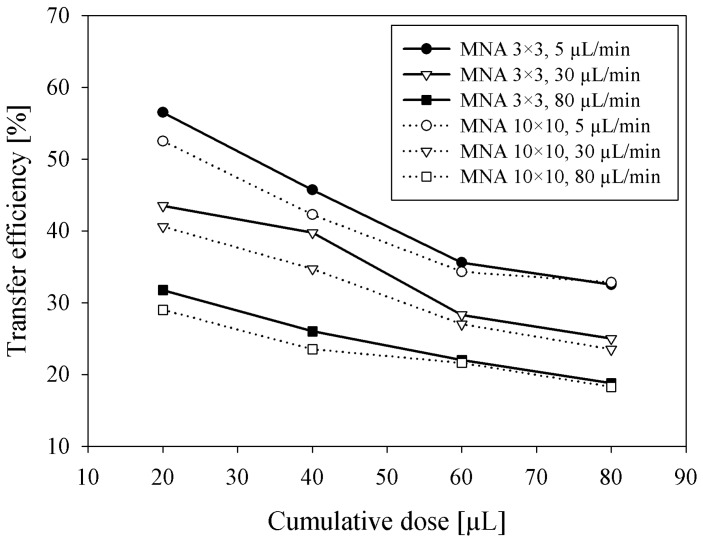
Transfer efficiency versus cumulative dose of saline solution delivered into the skin by 3 × 3 and 10 × 10 MNA at three different flow rates.

Besides the dependency of saline flow rate on transfer efficiency at a constant delivered dose, the influence of cumulative delivered dose on transfer efficiency was separately measured by adapting the delivery time for three specific flow rates. The aim of these experiments was to estimate the expected transfer efficiency of bolus insulin delivery where several insulin units (tens of microliters) should be delivered possibly in a short period of time. Measurement results shown in Figure 5 clearly show that the *η* is decreasing with a cumulative dose between 20 and 80 µL, presumably by the saturation effect due to low absorption of saline in viable epidermis. Smaller MNA (3 × 3) exhibited higher efficiency (3–5%) at lower doses compared to larger MNA (10 × 10). By increasing the cumulative dose, the difference between both MNA transfer efficiencies diminished, probably due to more accurate acquisition of residual saline left after higher doses. Accordingly, as shown previously in Figure 4, higher flow rates severely reduced the transfer efficiency.

#### 3.2.4. Backpressure versus Applied Force and Correlation with *η*

Total applied force on MNA and corresponding pressure distribution across the array is expected to influence the delivery performance in terms of microneedle insertion, underlying tissue compression and subsequent drug release through the microneedle array. Experiments were performed with 3 × 3 and 10 × 10 arrays. During saline delivery by syringe pump, the force applied on the MNA was varied between 1 N and 10 N. In order to reduce the error due to vaporization and to maintain constant force acting on MNA and the skin, the measurement duration was limited to 180 s. The delivered dose was kept the same for all measurements (90 µL).

We hypothesized that due to increased force, primarily the penetration depth will be increased and secondly the tightness between MNA chip perimeter and the skin will be improved. This would consequently allow the reaching of higher pressure of delivered substance without leaking and apparently improve transfer efficiency. 

**Figure 6:**
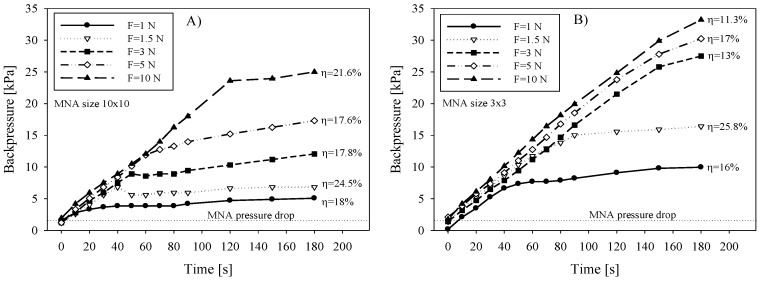
Delivery backpressure versus time during saline infusion into the skin as a function of applied force on the (**A**) 10 × 10 MNA and (**B**) 3 × 3 MNA. Saline flow rate was 30 µL/min.

Fluidic backpressure during saline delivery was measured by placing T-junctions in the delivery line and connecting the relative pressure sensor (HMIB001Z5, First Sensor AG, Berlin, Germany) pre-filled with saline. Figure 6A shows the measured backpressure data for 10 × 10 array during saline delivery into the skin. Independent measurements were performed by increasing the applied force and by keeping the flow rate constant (30 µL/min). The delivery of a substance by a syringe pump is pressure driven and is described by the backpressure given by the load, represented mainly by the tissue flow resistance. Two distinct slopes i.e., rise and stabilization of delivery backpressure versus time can be observed. Inflection point is an indication that the pressure rise was instantly changed due to additional flow conductive path. Initially, the pressure rose steeply, primarily due to adequate sealing between the chip perimeter and skin interface and partially due to limited stiffness of the tubing material. It could not be determined whether the leak between the microneedle and the skin appeared in this period. When the applied force cannot provide sufficient sealing due to increased backpressure (caused foremost by limited absorption in epidermis and a constant supply rate) the leak between the microneedle and the skin causes the surplus liquid to accumulate under the chip and finally spreads out. The calculated transfer efficiency (Figure 6A) on the basis of gravimetrical measurements was not found to be exactly proportional with the applied force, which could be partly attributed to a change in absorption properties of tissue due to compression.

In contrast to larger (10 × 10) arrays, a smaller array of 3 × 3 microneedles showed consistently higher backpressure due to higher applied pressure on MNA and consequently on the underlying tissue (Figure 6B). With respect to the applied force, higher values reached inflection point after longer time, meaning the MNA-skin interface sealing quality was increased due to higher pressure. However, the corresponding transfer efficiency showed in general lower values for smaller arrays and not entirely proportional to the applied force. Martanto et al. [41] reported that when excessive forces are applied, the tissue is more compressed, interstitial fluid is squeezed out and therefore porosity is decreased. Skin compaction thereby locally reduces flow conductivity and consequently also diffusivity. To avoid this, tissue relaxation by needle retraction was proposed [40,41,42]. It is important to note that in their study they were using longer, glass microneedle and rotational drilling device to assist the penetration. Our study showed that a proposed retraction procedure with a 10 × 10 array of 220 µm high microneedles could not be performed in a controllable manner and was not implemented in our in vivo tests.

Since the backpressure can be in general considered as a sum of the pressure drop due to microneedle hydraulic flow resistance and the tissue flow resistance, the contribution of MNA pressure drop *Δp* to the total measured backpressure was analytically evaluated. The pressure drop *Δp* as a function of flow rate *q* through the lumen of an individual microneedle is for laminar flow given by modified Bernoulli equation (Equation (2)). From the literature [43], the pressure drop *Δp* is expressed as a sum of pressure drop due to viscous shear force of Poiseulle flow inside the circular tube (first term) and the pressure drop due to inertia effect at entrance and exit (second term)

(2) $$\Delta p=\mu \frac{128}{\pi }\frac{ql}{d^{4}}+\rho \frac{8}{\pi^{2}}\frac{q^{2}}{d^{4}}\left(K_{1}+K_{2}\right)\;(\mathrm{Pa})$$

where *μ* is the dynamic viscosity and *ρ* is the density of liquid, *l* is the length and *d* is the diameter of microneedle lumen, respectively. For *K*_1_ and *K*_2_, values of 0.5 and 1 were adopted from the literature [43,44], for entrance and exit losses, respectively. Calculated pressure drop for open-end microneedles of 10 × 10 MNA was 1361 Pa, taking into account 50 µm lumen diameter and 400 µm long circular channel at chosen flow rate of 30 µL/min of saline. Contribution of the second term is only 49 Pa (3.6%) of the total pressure drop. The total pressure drop value was consistent with measured values shown in Figure 6 as an offset value (pressure just prior to pressing MNA on the skin at *t* = 0) depicted by dotted line. The maximal transfer efficiency *η* obtained from these experiments was between 24.5 and 25.8% for 10 × 10 and 3 × 3 array, respectively, which is in accordance with results in Figure 4A,B (*η* vs. flow rate) and in Figure 5 (*η* vs. dose). It is noteworthy that the highest efficiency was achieved for both arrays at 1.5 N of the applied force.

### 3.3. Insulin Control Delivery by Subcutaneous Route

**Figure 7:**
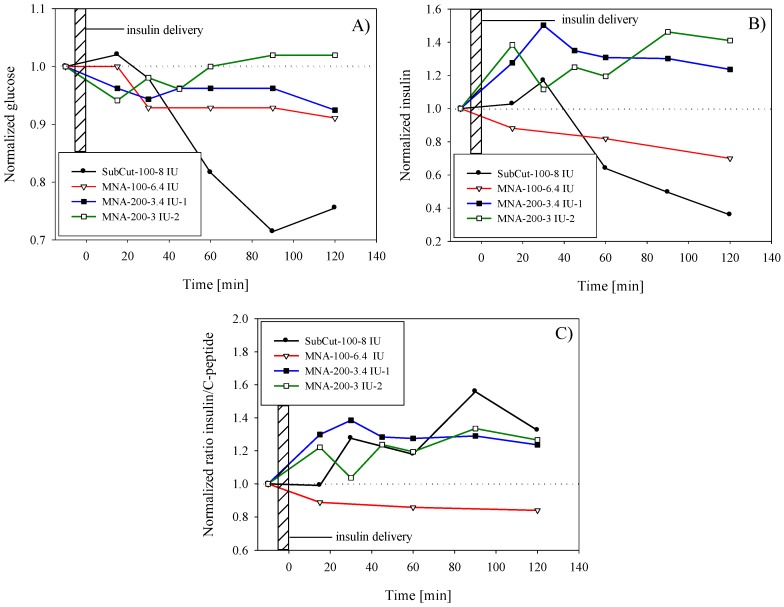
Time variation of (**A**) plasma glucose concentration after insulin delivery; (**B**) plasma insulin level after infusion of exogenous insulin; (**C**) serum insulin to C-peptide ratio. Results are normalized by the preinfusion values (baseline).

Subcutaneous delivery was performed by using 8 IU (international units) of fast-acting insulin (NovoRapid^®^, 100 IU/mL, Novo Nordisk Inc., Mississauga, ON, Canada). Insulin was administered subcutaneously using an insulin pen in order to obtain the reference response of healthy human subject. Estimated penetration depth was between 5 and 6 mm, thus reaching subcutis region. As shown by glucose level monitoring in Figure 7A (black circles), continuous decrease of blood glucose level started only after 15 min, reaching a minimum after 90 min (72% of preinfusion level). This was followed by a recovery phase in order to reach homeostasis. For the presentation purpose, obtained values for glucose and insulin were normalized by the preinfusion values (blood samples taken 10–15 min prior to insulin infusion). Results of serum insulin analyses are presented in Figure 7B. An increase of insulin and an apparent lag of insulin peak (20% above the preinfusion level) due to exogenous insulin could be observed in the first 30 min and can be explained in terms of insulin absorption and action phases. Even the subcutaneous route was reported to be affected by the time delay in absorption with respect to e.g., intravenous route [45].

### 3.4. Microinjection of Insulin through Hollow MNA 

The first experiment with insulin delivery through hollow MNA (Figure 1B,C) was performed on the same human subject with the aid of the fixture shown in Figure 2. A syringe pump was used to provide pressure-driven flow with a controlled flow rate. The targeted dose of insulin (NovoRapid^®^, 100 IU/mL) was 8 IU to enable comparison with subcutaneous delivery. The insulin flow rate was set at 30 µL/min, primarily to closely mimic the bolus mode of insulin delivery as performed previously by the subcutaneous route. Another reason for higher flow rate was to reduce the delivery time and to avoid disturbances due to arm movement during the longer delivery period.

After delivering the targeted amount, the device was allowed to stay in place for 1 min, thus slowly releasing the backpressure. MNA was then removed from the skin, and the residual insulin was collected from the skin surface and weighted. The determined efficiency with gravimetric method described earlier showed that the actual dose was 6.4 IU, i.e., lower than the targeted dose. Also the following experiments revealed similar deviation from the targeted dose.

After performing blood analyses, the determined effect on glucose level was less pronounced compared to subcutaneous delivery. As shown in Figure 7A (white triangles), the glucose level started to decrease after a 15 min delay. After 30 min, the glucose level was decreased by 7% with respect to the preinfusion level, and then remained for up to 2 h within the same range. It is noteworthy that in contrast to subcutaneous delivery, the MNA method did not show tendency of establishing initial glucose level even after 120 min that could indicate slow diffusion of exogenous insulin, which is still taking place after 120 min. The increase of insulin level in blood due to infusion of exogenous insulin was not detected; on the contrary, the level monotonically decreased with time. Similar but more pronounced insulin decrease (after reaching maximum after 30 min) was observed also in the case of subcutaneous delivery.

In order to amplify the effect of glucose drop by infusion of exogenous insulin by MNA and overcome the constraints such as limited transfer efficiency and maintaining the short delivery time, a more-concentrated rapid-acting insulin was selected for the next experiment. By using more-concentrated (2×), fast-acting insulin Humalog^®^ 200 IU/mL (Eli Lilly and Company Ltd., Toronto, ON, Canada) we could therefore halve the intake time for delivering the equal quantity of insulin units, thus mimic the bolus type of infusion. Two separate tests with the same target dose (8 IU) were performed within a 3-month period on the same human subject as abovementioned.

The results of the first test in Figure 7A (black squares) show modest but instant decrease in glucose in the range of 5–7%. In contrast, serum insulin level (Figure 7B) was significantly increased with respect to preinfusion level and accounted for nearly 50% at peak value (at *t* = 30 min after the completed infusion). It is believed that this increase was mainly due to successful infusion of exogenous insulin. Furthermore, the insulin peak coincided with glucose drop at *t* = 30 min. Since the subject’s preinfusion glucose was stable and within the limits of normal (5.3 mmol/L), it is difficult to explain why a surplus of insulin was not acting on further glucose decrease as in the case of subcutaneous infusion. One possible explanation could be insufficient penetration depth of microneedles; however, we were not able to monitor this parameter within experiments. In this test, gravimetrically determined dose was 6.8 IU, which was about 15% lower than targeted dose.

The second test (white squares) with Humalog^®^ 200 IU/mL was repeated under the same parameters as above and the results are shown by open square symbols in Figure 7A,B for glucose and insulin, respectively. Despite certain variations in resulting values in the second test, results show rather repeatable behavior in terms of glucose drop and in terms of increased amount of insulin. By determining the transfer efficiency after the test (25%), it was shown that only 6 IU was delivered. Glucose level drop accounted for 5% after 15 min and then slowly returned to preinfusion value. Compared to the first test, in the second test the glucose minimum and peak of insulin occurred more rapidly. It was expected that in the case of the transdermal route the lag would be even more pronounced, however no particular difference was observed compared to the subcutaneous route.

The amount of insulin was in both tests with more-concentrated Humalog^®^ 200 IU/mL well above the preinfusion level, which confirmed the successful delivery of insulin; however, due to slow absorption and diffusion through the viable epidermis layer, the action differs significantly from the quick and deeper subcutaneous insulin delivery.

By introducing the insulin to the C-peptide ratio, normalized by the preinfusion ratio (Figure 7C) it became more evident that successful delivery was performed. Increase of insulin to C-peptide ratio above the preinfusion ratio proved that the increase of total serum insulin was in the first place a consequence of exogenous insulin infusion and not due to secretion of endogenous insulin. The subcutaneous delivery and delivery by MNA with more-concentrated insulin show similar behavior in contrast to the infusion of standard (100 IU/mL).

## 4. Discussion

### 4.1. Skin Penetration Mechanism by Hollow MNA

In humans, the outermost, lipophilic SC layer is the skin’s major barrier for hydrophilic insulin delivery. Besides, it has been determined previously that due to viscoelastic properties of the skin the entire microneedle shaft will not penetrate the skin. The depth of penetration was estimated by Roxhed et al. [39] to be one half of the microneedle height, while other study reported only 10–30% of the microneedle height penetrated the skin [29]. According to Mukerjee et al. [22] and Roxhed et al. [39] sharp, solid microneedles push the SC corneocytes apart during penetration, thereby causing very little tissue damage. In the case of hollow microneedles the skin tends to bend and fold around the needle tip to a depth greater than the needle shank height.

The skin deformation mechanism during penetration of a sharp object has been studied by several authors [46,47,48,49]. The model of Crichton [49] assumes that during the insertion of a sharp solid microneedle the skin is put in a higher tension and therefore reduces the insertion force. For hollow microneedle penetration, a model relies on the assumption that the needles behave like a rod [47] and does not account for the behavior of the skin beneath the lumen area.

Besides the successful penetration through the SC, another important aspect is a passage continuity for the delivered drug, which may be obstructed by clogging the lumen of the hollow microneedle. One of concerns was that sharp a circular microneedle apex would cut the SC, which would then enter the lumen during penetration and eventually obstruct the flow path. Due to the lipophilic nature of the SC layer and due to insufficient penetration of microneedles, this compressed skin layer inside the lumen would represent a severe barrier for hydrophilic substances to diffuse through and the drug passage would be severely obstructed. However, during this study we found no optical evidence of a sharp tip cutting through the skin or observe any clogging of microneedle lumens after removal. Therefore, we assume that displacement of skin via crack propagation through brick and mortar structures as proposed by some authors [23,48] is the most viable mechanism of hollow microneedle penetration through the SC.

### 4.2. Saline Delivery into the Skin 

Saline infusion into the skin revealed the importance of delivery parameters and their influence on the transfer efficiency. As shown in Figure 4, results obtained by saline infusion showed significant reduction in transfer efficiency by increasing the saline flow rate. In the 1–3 μL/min range, the transfer efficiency exceeded 90% at best, however, it exponentially decreased toward 20% for flow rates exceeding 50 μL/min.

Compared to 10 × 10 MNA, the 3 × 3 MNA showed higher transfer efficiency most probably due to the fact that penetration of the skin by large microneedle array is less effective as a consequence of the pressure distribution across several microneedles for the same range of applied force.

One of the major concerns in transdermal delivery by MNA is the control of the delivered amount of drug, which can be affected by unpredicted leaks due to various reasons (movement, sweat pores, hair obstructions, damaged microneedle, variation of applied pressure on MNA during delivery period, etc.). Each of them or their combination can severely affect the accuracy of delivering the required amount of drug.

Leak-free delivery is very difficult to obtain due to increased pressure drop at microneedle-skin interface exhibiting large flow resistance imposed by epidermal sub layers. When the applied force on a MNA is high (range 5–10 N), perimeter sealing of the Si chip is improved and therefore inlet pressure can reach a higher value without apparent leak. When low force (*F* < 0.5 N) was applied to MNA, the leak was observed to increase due to insufficient perimeter sealing. Since the MNA is pressed to the skin with defined force, the distribution of pressure that provides perimeter sealing is mainly defined by the flexibility of skin. 

To summarize, when the amount of supplied substance surpasses the amount that can be delivered and simultaneously absorbed in the viable epidermis, a leak occurs at the microneedle-skin interface due to pressure-driven supply. Finally, it exceeds the pressure ensuring the sealing, which is given by the applied force on the MNA. In this case, a lateral leaking path is formed between the MNA chip edge and the skin and the equilibrium is established. Hereon, the amount of supplied liquid is a sum of liquid that the tissue is capable of absorbing and redistributing and the surplus liquid remains on the skin surface. This is clearly shown in Figure 6 as inflection point. It was shown that increasing the applied force has not increased the transfer efficiency, which is noteworthy, but has severely increased the patient’s discomfort.

Some additional factors that might enhance the transfer efficiency were also taken into account. Preconditioning the skin prior to MNA application by additional drying or moistening, striping the dead layers of SC by scotch tape or abrasive SC removal, did not prove efficient in terms of improved transfer efficiency. Furthermore, we considered the application at different anatomical sites, which might affect the transfer efficiency primarily due to the thickness of SC and viable epidermis as well as due to mechanical properties of supporting tissue. Locations such as upper arm, dorsal and ventral side of forearm, arm wrist, upper leg and abdomen position were selected for comparison. No measurable difference was determined taking into account the tolerance limits of measurement method. 

### 4.3. Insulin Delivery into the Skin

By applying only optimized parameters determined in saline infusion experiments the in vivo tests with insulin showed consistent behavior. Subcutaneous reference delivery of 8 IU to a healthy human subject showed significant drop of glucose (30% drop reaching minimum at 90 min). By infusion of 6.4 IU of insulin (Novorapid 100 IU/mL) with aid of MNA 10 × 10, decrease of glucose level accounted only for 5–10% and was nearly maintained during a 120 min period. The determined transfer efficiency was 27%, which was in accordance with saline experiment for the flow rate of 30 μL/min (25–40%). In this case, serum insulin analyses did not confirm the uptake of exogenous insulin (Figure 7B, open triangles), neither has the insulin to C-peptide ratio increased (Figure 7C, open triangles). Infusion of more-concentrated insulin (200 IU/mL) through hollow MNA resulted in significant increase of insulin in blood in comparison to previously used 100 IE/mL insulin and for delivering nearly the same targeted dose (6.8 and 6.4 IU. respectively). Delivery time was halved in the case of 200 IU/mL insulin, approaching the bolus type of delivery. The gravimetrically determined dose after the first infusion test of concentrated insulin accounted for 6.8 IU (with transfer efficiency of 28.3%) and for the second only 6 IU (transfer efficiency of 25%). The protocol was maintained strictly the same in all three experiments and transfer efficiencies were repeatable (25–28%), which was in agreement with tolerances and expected transfer efficiencies presented previously. Despite the fact that no hypoglycemic response was detected by the healthy subject, the increase of insulin (40–50%) as shown by the two experiments in Figure 7B can be related entirely to the enhanced absorption and diffusion of more-concentrated insulin compared to standard concentration (100 IU/mL). It is well known that the driving force for enhanced uptake of insulin, reaching the systemic circulation can be in this case larger concentration gradient, which could play a major role in diffusion process. In general, flux *φ* through the skin layers is given by the expression (Equation (3)):

(3) $$\varphi =-P(c_{2}-c_{1})$$

where *P* is the skin permeability (of the layers, which the drug has to traverse before entering the circulatory system) for a given substance at a given temperature and *c*_2_ − *c*_1_ is the difference in concentration of the substance across the membrane for the direction of flow (from *c*_1_ to *c*_2_). Total transdermal diffusion processes are complex because skin represents inhomogeneous anisotropic media and diffusion coefficient varies within sublayers [50] and were not the subject of this study.

One of the important conclusions of this case study was that a bolus type of insulin infusion can be accomplished with limited transfer efficiency (bioavailability), which is directly related to the required high flow rate. To increase the transfer efficiency, it would be mandatory to decrease the insulin flow rate, indicating that the method would be more appropriate for covering basal needs of the patient with corresponding type of insulin. 

The classical insulin administration into the abdominal subcutaneous layer (adipose tissue) allows a slow release of insulin for the required therapeutic effect at the expense of low patient compliance due to painful administration. In our case, Figure 7B,C showed the relative constant insulin blood levels after MNA-facilitated administration, compared with the fluctuating insulin levels after SC administration. Furthermore, we can discuss the difference between the bolus administration and multiple doses regimen: the relatively constant plasma concentration profile after MN administration is close to a “steady state” after a multiple doses regimen or to a slow-release drug type of formulation. Therefore, this profile mimics the stable absorption and distribution of insulin for a constant plasma concentration with potential therapeutic benefits.

Further work will be undertaken to improve the transfer efficiency by increasing the microneedle height and related skin penetration depth, while still retaining the microneedles robustness, safety and most of all the patient’s compliance.

## 5. Conclusions

Design and fabrication of high-aspect-ratio hollow silicon MNA for transdermal drug delivery for microinjection of insulin together with a delivery carrier method are reported. Delivery experiments were optimized using MB for the leaking test and also by in vivo infusion of saline in order to provide quantitative results of transfer efficiency through the skin. Finally, delivery of insulin was performed and characterized by blood analyses of glucose, insulin and c-peptide.

Presented gravimetric method enabled quantification of the transfer efficiency and provided fast and relatively accurate evaluation as compared to analysis of body liquids, which is time consuming and demanding. Transfer efficiency was found to decrease exponentially by increasing the saline flow rate and delivered dose. Increase of applied force on the MNA during delivery showed minor influence on transfer efficiency. Preconditioning of the skin and choice of delivery location has not provided any substantial improvement in transfer efficiency. Based on the above results, the dominant factor limiting the transfer efficiency was found to be limited absorption and diffusion process within the viable epidermis.

Results of in vivo insulin delivery proved successful infusion of fast-acting insulin as shown by blood analyses. Compared to subcutaneous delivery of nearly equal infusion dose, MNA delivery showed a less significant drop of glucose level, but a noteworthy increase of serum insulin (40–50%), attributed primarily to more effective delivery of concentrated (200 IU/mL) exogenous insulin. The proposed method was able to deliver insulin and to ensure a relatively constant plasma concentration. Regarding patient compliance, MNA delivery method proved to be painless and not causing any skin irritation or inflammation at delivery sites.
